# Global analysis of erythroid cells redox status reveals the involvement of Prdx1 and Prdx2 in the severity of beta thalassemia

**DOI:** 10.1371/journal.pone.0208316

**Published:** 2018-12-06

**Authors:** Karen S. Romanello, Karina K. L. Teixeira, João Pedro M. O. Silva, Sheila T. Nagamatsu, Marcos André C. Bezerra, Igor F. Domingos, Diego A. P. Martins, Aderson S. Araujo, Carolina Lanaro, Carlos A. Breyer, Regiane A. Ferreira, Carla Franco-Penteado, Fernando F. Costa, Iran Malavazi, Luis E. S. Netto, Marcos A. de Oliveira, Anderson F. Cunha

**Affiliations:** 1 Universidade Federal de São Carlos (UFSCar), Departamento de Genética e Evolução, São Carlos, Brazil; 2 Universidade de Campinas (UNICAMP), Departamento de Genética, Evolução e Bioagentes, Campinas, Brazil; 3 Universidade Federal de Pernambuco (UFPE), Departamento de Genética, Pernambuco, Brazil; 4 Fundação de Hematologia e Hemoterapia do estado de Pernambuco (HEMOPE), Pernambuco, Brazil; 5 Hemocentro da Universidade de Campinas (UNICAMP), Campinas, Brazil; 6 Universidade Estadual Paulista (UNESP)–Campus Litoral Paulista, São Vicente, Brazil; 7 Universidade de São Paulo (USP), Departamento de Genética, Biologia Evolutiva, São Paulo, Brazil; University of Alabama at Birmingham, UNITED STATES

## Abstract

*β*-thalassemia is a worldwide distributed monogenic red cell disorder, characterized by an absent or reduced beta globin chain synthesis. The unbalance of alpha-gamma chain and the presence of pathological free iron promote severe oxidative damage, playing crucial a role in erythrocyte hemolysis, exacerbating ineffective erythropoiesis and decreasing the lifespan of red blood cells (RBC). Catalase, glutathione peroxidase and peroxiredoxins act together to protect RBCs from hydrogen peroxide insult. Among them, peroxiredoxins stand out for their overall abundance and reactivity. In RBCs, Prdx2 is the third most abundant protein, although Prdxs 1 and 6 isoforms are also found in lower amounts. Despite the importance of these enzymes, Prdx1 and Prdx2 may have their peroxidase activity inactivated by hyperoxidation at high hydroperoxide concentrations, which also promotes the molecular chaperone activity of these proteins. Some studies have demonstrated the importance of Prdx1 and Prdx2 for the development and maintenance of erythrocytes in hemolytic anemia. Now, we performed a global analysis comparatively evaluating the expression profile of several antioxidant enzymes and their physiological reducing agents in patients with beta thalassemia intermedia (BTI) and healthy individuals. Furthermore, increased levels of ROS were observed not only in RBC, but also in neutrophils and mononuclear cells of BTI patients. The level of transcripts and the protein content of Prx1 were increased in reticulocyte and RBCs of BTI patients and the protein content was also found to be higher when compared to beta thalassemia major (BTM), suggesting that this peroxidase could cooperate with Prx2 in the removal of H_2_O_2_. Furthermore, Prdx2 production is highly increased in RBCs of BTM patients that present high amounts of hyperoxidized species. A significant increase in the content of Trx1, Srx1 and Sod1 in RBCs of BTI patients suggested protective roles for these enzymes in BTI patients. Finally, the upregulation of *Nrf2* and *Keap1* transcription factors found in BTI patients may be involved in the regulation of the antioxidant enzymes analyzed in this work.

## Introduction

Increased reactive oxygen species (ROS), such as superoxide anion (O_2_^•-^), hydrogen peroxide (H_2_O_2_) and hydroxyl radical (HO^•^), have been associated with the aggravation of several diseases, including hemolytic anemia, such as beta thalassemia (βthal) [1, 2]. This disease is caused by a quantitative alteration of beta globin synthesis and can be genetically classified in two types: βthal-β^0^ when the synthesis of beta globin is absent, and βthal-β^+^ when there is a reduction in synthesis’ rate, leading to a lower production of hemoglobin that causes various degrees of anemia [3, 4]. More than 200 mutations within the beta-globin gene were associated with βthal; in Brazil, the mutations most commonly associated with the disease are β^0^ IVS-I-1 (G→A), β^+^ IVS-I-6 (T→C) and β^0^39 (C→T). Clinically, βthal can be classified as minor (individuals are usually asymptomatic), major (severe anemia, with dependence on regular blood transfusions) or intermedia (formed by clinical phenotypes between minor and major phenotypes) [5, 6]. In βthal, mainly in the intermedia and major phenotypes, the cellular environment is extremely pro-oxidative, mostly due to the excess of unpaired alpha globin chains. These chains can form unstable tetramers that precipitate and release the heme group and iron upon oxidation. This event contributes to the formation of HO^•^ by chemical reactions of the Fe with O_2_^•-^ and H_2_O_2_ such as Fenton. The HO^•^ can also be formed by the Harber Weiss direct reaction between O_2_^•-^ and H_2_O_2_ [7, 8]. These ROS leads to damage of red blood cells (RBCs), membrane components such as band 3, spectrin, protein 4.1, and ankyrin, proteins that contributes to the hemolysis [9]. Since RBCs can access various organs and tissues, the hemolysis may also contribute to oxidative damage in other tissues [10–12].

To avoid HO^•^ formation, the cells developed different defense mechanisms against ROS. Superoxide dismutase (Sod) which converts the superoxide anion (O_2_^•-^) into molecular oxygen (O_2_) and hydrogen peroxide (H_2_O_2_) is the primary defense [13].

H_2_O_2_ can be further decomposed by three different pathways that act simultaneously and are catalyzed by the enzymes catalase (Cat), glutathione peroxidase (Gpx) and peroxiredoxins (Prdxs). Prdxs stand out for their abundance and reactivity with their substrates [14]. They are able to catalyze the reduction of hydrogen peroxide, organic hydroperoxides and peroxynitrite, using a highly reactive cysteine residue present at its catalytic site called peroxidatic cysteine (Cys_P_) [15].

In humans, six isoforms of Prdxs (Prdx1-6) have been described and their subdivision is based on catalytic mechanism and number of cysteines involved in the enzymatic catalysis in typical 2-Cys Prdxs (Prdx1-4), 2-Cys atypical Prdx (Prdx5) and 1-Cys Prdx (Prdx6) [14–17]. These enzymes are widely distributed in the cell and are present in the cytosol (Prdx1, Prdx2, and Prdx6), mitochondria (Prdx3 and Prdx5), endoplasmic reticulum (Prdx4), nucleus (Prdx1) and even in association with membranes (Prdx1 and Prdx2) [18]. After hydroperoxide reduction, the catalytic cysteine residue is oxidized. The Prdx reduction is frequently performed by the Trx system, which comprises the enzymes thioredoxin (Trx1) and thioredoxin reductase (TrxR1), by using electrons from NADPH. Under high levels of hydroperoxides, the 2-Cys Prdx Cys_P_-SOH can react with another H_2_O_2_ molecule and become hyperoxidized to cysteine sulfinic acid (Cys_P_ -SO_2_H) or sulfonic acid (CysP-SO_3_H). The Cys_P_ hyperoxidation is linked to the loss of peroxidase activity but some studies point to a gain of function as molecular chaperone [19]. When in Cys_P_-SO_2_H form, activity can be reestablished through reduction by the enzyme sulfiredoxin (Srx), in an ATP-dependent reaction. However, hyperoxidation to Cys_P_-SO_3_H, leads to permanent catalytic inactivation [19–21].

In mature erythrocytes, only the cytosolic isoforms Prdx1, Prdx2, and Prdx6 are found, since this cell type does not possess organelles [22]. Among these isoforms, Prdx2 is the third most abundant protein and one of the leading cytoprotective agents; it is a sensitive real-time marker of systemic neutrophil activation and, consequently, of inflammation activation by oxidative stress [23, 24]. Although several studies have demonstrated the importance of Prdxs for the differentiation and maintenance of erythrocytes in hemolytic anemia [22, 25–29], there are few studies regarding the role of these enzymes in βthal intermedia. Therefore, this study aimed to evaluate the expression pattern of these antioxidant enzymes and their enzymatic reductants in reticulocytes and RBCs of BTI patients, which have significantly elevated levels of ROS, and to compare to reticulocytes and RBCs of healthy individuals. The involvement of *Nrf2/Keap1* transcription factor complex in the regulation of these proteins was also evaluated. In addition, 2-Cys Prdxs production and hyperoxidation were compared among RBCs of healthy individuals and BTI and BTM patients. This study presents, for the first time, an overview of redox status, providing evidences that Prdx1 cooperates with Prdx2 in the antioxidant pathways of erythroid cells from patients with βthal intermedia.

## Material and methods

### Patients and controls

Patients previously diagnosed by the Hematology and Hemotherapy Foundation of the Pernambuco State (HEMOPE Foundation) with BTI, homozygous for the IVS-I-6 (T → C) mutation, were enrolled in this study. Samples of control subjects were collected from healthy volunteers. The Ethics Committee from the Federal University of São Carlos and Federal University of Pernambuco approved this study under the reference number CAAE: 31939814.1.1001.5504. The patients signed a written informed consent before their inclusion in this study.

A total of 15 patients with BTI from 8 to 63 years of age (46.4 ± 13.63) were analyzed, being 8 females and 7 males. The analyzed controls totalized 16 healthy individuals, with ages ranging from 22 and 42 (28 ± 1.67), being 8 females and 8 males. In addition, samples of 8 BTM patients were used for comparative purposes. These individuals ranged from 3 to 33 years of age (19.75 ± 15.08) and consisted of 5 males and 3 females, presenting the following genotypes: IVS-I-6 (T → C) / IVS-I-5 (G → C) (3 individuals), IVS-I-6 (T → C) / IVS-I- 1 (G → A) (1 individual), IVS-I- 1 (G → A) / IVS-II- A → G) (1 individual), CD39 (C→T) (2 individuals), and one of them was not genotyped. To avoid artefacts of the Prdx1/Prdx2 hyperoxidation related to circadian cycle [30, 31], all blood samples were collected between 7 and 9 am. Subject criteria for inclusion and exclusion were based on clinical diagnosis for the disease. Hematologic data of the patients at the time of the sample harvest are presented in Table 1.

**Table 1:** Hematologic data of beta-thalassemia patients.

Parameters	Beta thalassemia intermedia n = 15	Beta thalassemia major n = 8
**RBC (10**^**6**^**mm**^**3**^**)**	3.77 ± 0.65	3.07 ± 0.46
**Hb (g/dL)**	7.6 ± 0.71	6.5 ± 1.82
**VCM (fL)**	66.6 ± 8.82	74.1 ± 1.32
**Ret (%)**	7.1 ± 3.69	4.3 ± 1.8
**Hct (%)**	25.2 ± 1.79	22.5 ± 3.72
**HbF (%)**	10.5 ± 5.91	38.22 ± 40.05
**HbA (%)**	82.7 ± 5.38	57.7 ± 39.86
**HbA**_**2**_**(%)**	6.8 ± 1.16	2.9 ± 0.21
**WBC (10**^**3**^**mm**^**3**^**)**	10.738 ± 5.984.66	14.300 ± 3.591.65

RBC, red blood cell; Hb, hemoglobin; MCV, mean corpuscular volume; Ret, reticulocyte; Hct, hematocrit; HbF, hemoglobin fetal; HbA, hemoglobin A, HbA_2_, hemoglobin A_2_; WBC, white blood cell. Data are presented as a mean and standard deviation. All BTM patients are under regular transfusion and iron chelation therapy. BTI patients are under non-regular transfusion and without iron chelation therapy.

### Red cell separation

Peripheral blood samples were collected in a tube with sodium citrate containing N-ethylmaleimide (NEM; 200 mM). They were then centrifuged for plasma and buffy coat removal. Cells were washed 3 times in PBS 1× (buffered phosphate saline, pH 7.4) and resuspended in 1 mL of PBS for counting in a Cell-Dyn 1700 automatic counter (Abbott Diagnostics, Lake Bluff, Illinois, USA). The final concentration was adjusted to 4 × 10^8^ cells/mL. For determination of ROS production, the red cells were counted in a Neubauer chamber and resuspended at a concentration of 1 × 10^6^ cells/mL.

### Isolation of neutrophils and mononuclear cells from peripheral blood

All blood samples from control subjects and patients were collected in lithium heparin tubes (9 mL) and separated with Ficoll-Hypaque (Sigma-Aldrich, St. Louis, Missouri, USA) at densities of 1.119 g/L and 1.077 g/L. After separation of mononuclear cells and granulocytes, the contaminating red blood cells were lysed with lysis buffer (0.144 M NH_4_Cl; 0.01 M NH_4_HCO_3_) and washed again in PBS. To determine the production of ROS, mononuclear cells and granulocytes were counted in a Neubauer chamber and resuspended at a concentration of 1 × 10^6^ cells/mL.

### Determination of the production of reactive oxygen species (ROS)

RBC, granulocytes and mononuclear cells from patients and controls were incubated with 0.5 μL of 2,7-Dichlorodihydroflurane-diacetate (DCFH-DA) (Invitrogen-Thermo Fisher Scientific, Waltham, Massachusetts, USA). The ROS production was analyzed by flow cytometry (FACS-Calibur, Becton-Dickinson, Immunofluorometry systems, Mountain View, California, USA) with an acquisition of 10,000 events using the CellQuest program for analysis of mean fluorescence intensity (MFI).

### Separation of Reticulocytes

Peripheral blood samples were collected in tubes with EDTA (ethylenediaminetetraacetic acid) and centrifuged for plasma removal. The erythrocytes were lysed with red blood cells lysis solution (0.144 M NH4Cl; 0.01 M NH4HCO3) and centrifuged for the collection of the supernatant, which was homogenized with 1/10 volume of a Sucrose/KCl solution (1.5 M Sucrose, 0.15 M KCl). After further centrifugation, the supernatant containing only reticulocytes was treated with 10% acetic acid and then centrifuged. The pellet was resuspended in 1 mL of Trizol (Invitrogen-Thermo Fisher Scientific, Waltham, Massachusetts, USA). The efficiency of the reticulocyte separation and a possible contamination with leukocytes was determined by microscopic observation after isolation using panotic and brilliant blue crezil dyes (S1 Fig)

### RNA extraction and quantitative real-time PCR (RT-qPCR) procedures

Reticulocyte RNA was extracted by the Trizol method (Invitrogen-Thermo Fisher Scientific, Waltham, Massachusetts, USA) according to manufacturer's instructions. A total of 1 μg of RNA was treated with DNAseI (Invitrogen-Thermo Fisher Scientific, Waltham, Massachusetts, USA) and was reverse transcribed with High Capacity cDNA Reverse Transcription kit (Applied Biosystems-Thermo Fisher Scientific, Waltham, Massachusetts, USA). RT-qPCR was conducted using Power Sybr Green PCR Master Mix (Applied Biosystems-Thermo Fisher Scientific, Waltham, Massachusetts, USA) on StepOne Plus Real Time PCR System (Applied Biosystems-Thermo Fisher Scientific, Waltham, Massachusetts, USA). All the primers were designed using OligoAnalyzer 3.1 (Integrated DNA Technologies, Coralville, Iowa, USA) and are listed in S1 Table. The concentration of primers was optimized prior to efficiency curve reaction and their efficiency ranged from 95%-105%. Relative fold change in mRNA quantity was calculated according to 2^(−ΔΔCt)^ method and all values were normalized to the expression of the human β-actin (BAC) gene [32].

### Protein extraction and western blot analysis

Peripheral blood samples were centrifuged at 3,000 rpm, for 10 minutes at 4°C, after which plasma and buffy coat were discarded. For protein extraction, the red cells were lysed with extraction buffer: EDTA (10 mM), Trizma base (100 mM), Na_4_P_2_O_7_·10H_2_O (10 mM), NaF (100 mM), Na_3_VO_4_ (10 mM), PMSF (2 mM), Aprotinin (0.1 mg/mL), and Triton (1%) with the addition of 1× Complete Mini protease inhibitor (Roche Applied Science). Then, the same volume of buffer was added to the red blood cell pellet. Samples were incubated for 40 minutes on ice with vigorous shaking every 5 minutes. After centrifugation at 12,000 *g* for 20 minutes at 4°C, the supernatant containing lysed erythrocytes was transferred to a new tube, and the proteins were quantified by the Lowry method [33]. 50 μg of protein from each sample were resolved in a 12% (w/v) SDS–PAGE and transferred to a nitrocellulose membrane (Bio-Rad, Hercules, Calif., USA). All primary antibodies were used following manufacturer’s instructions: anti-Prdx2 (dilution 1:5,000, Abnova #H00007001-M01); anti-Prdx6 (dilution 1:5,000, Abnova #H00009588-M01) and anti-Trx1 (dilution 1:2,500, Abnova #H00007295-M01); anti-Prdx1 (dilution 1:4,000, Cell Signaling #8499); anti-catalase (dilution 1:5,000, Cell Signaling #12980); anti-Gpx1 dilution (1:4,000, Abcam #ab22604) and anti-Sod1 mAb (dilution 1:5,000, Abcam #ab16831); Anti-Srx1 polyclonal rabbit (dilution 1:2,500, Proteintech Group Inc #14273-1-AP); anti-Trxr1 mAb (dilution 1:2,000, AbFrontier Co #LF-PA0023); anti-GAPDH polyclonal rabbit dilution (1:10,000, EMD Millipore #ABS16). The latter was used as loading control in the experiment. For Prdx hyperoxidation analysis, we used anti-Prx-SO_3_ (dilution 1:2500, AbFrontier Co #LF-PA0004). It was diluted in TBST containing 3% skimmed milk and incubated on a rocking platform for 16 hours at 4°C. Antibodies were detected using a peroxidase (HRP)-conjugated second antibody (GE Healthcare—Little Chalfont United Kingdom). Chemoluminescent detection was obtained by using ECL Prime Western Blot detection kit (GE Healthcare–Little Chalfont United Kingdom). Membranes were exposed using ChemiDoc equipment (Bio-Rad—Hercules, California, EUA) to generate the images.

### Statistical analysis

Data are presented as mean with the Standard Error of the Mean (SEM). They were compared with the Mann-Whitney U-test. Values of p <0.05 were considered statistically significant. Statistical analysis was based on nonparametric parameters according to Quinn et al [34].

## Results

### ROS production is increased in different types of blood cells

**Fig 1:**
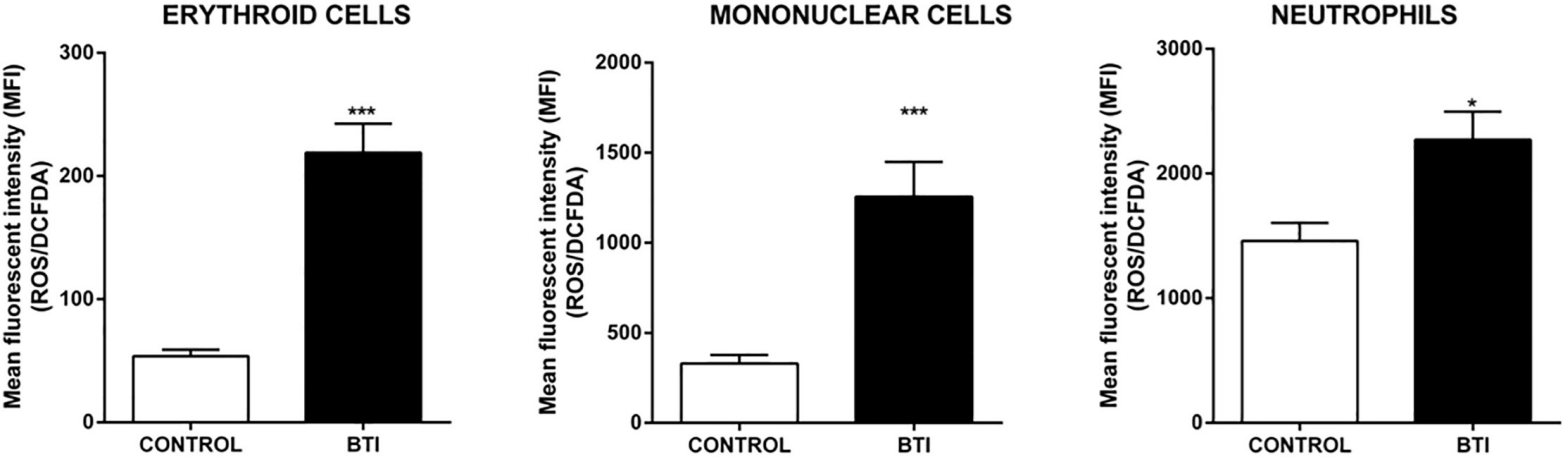
ROS production was increased in different cell types of patients with β Thalassemia intermedia. Analysis of DCFDA fluorescence intensity, corresponding to the level of ROS production in erythroid, mononuclear and neutrophils cells. The results are expressed as mean (±SEM) fluorescence intensity (MFI) emitted by the analyzed cells of control and BTI patients. Statistical difference: (*) p < 0.05 and (***) p < 0.001.

Increased ROS generation plays an important role in the pathophysiology of βthal, contributing significantly to the hemolytic processes found in this disease. Therefore, we analyzed the formation of DCFDA fluorescent products in the RBC, neutrophils and mononuclear cells from peripheral blood of patients with BTI (n = 13) and healthy individuals (Control; n = 15). A significant increase in the oxidative state was observed in all of the analyzed cell types from BTI patients (Fig 1).

### Transcription of redox enzymes are altered in reticulocytes of BTI patients

**Fig 2:**
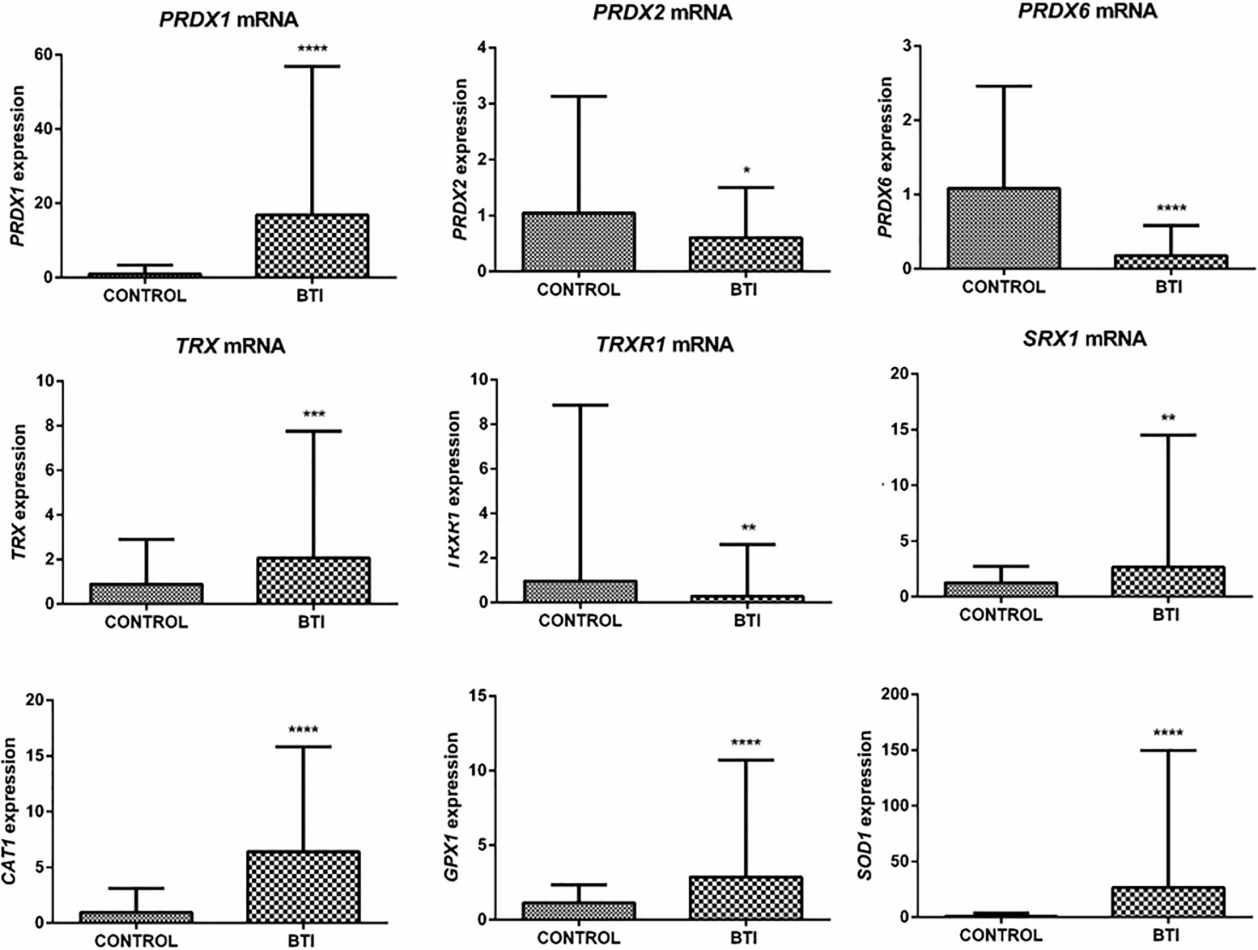
Transcription analysis of redox gene expression responsible for the production of antioxidant enzymes in reticulocytes of control individuals and BTI patients. RT-qPCR was carried out using the primers for each gene described in S1 Table. mRNA abundance for each gene was normalized to *Bac*, except for *TrxR1*, where *Hprt1* was used as the endogenous reference. Results are presented as mean with standard error (± SEM). Statistical significance: (*p < 0.05), (**p < 0.001), (***p < 0.0001).

The high ROS levels observed in BTI evidenced the importance of the enzymatic antioxidant system for the maintenance and survival of red blood cells in the circulation. Using RT-qPCR, we evaluated the transcription levels of Prdxs and the physiological reductants of 2-Cys Prdxs, as well as the other enzymatic antioxidants in reticulocytes of BTI and healthy subjects. Although anucleate, reticulocytes still present recently transcribed mRNA remains, allowing the use of this technique. The transcription of all analyzed antioxidants was modulated in BTI. *Prdx1*, *Trx1*, *Srx1*, *Cat1*, *Gpx1* and *Sod1* genes showed a significant increase in their transcription levels when compared to healthy individuals. On the other hand, mRNA levels of *Prdx2*, *Prdx6* and *TrxR1* were significant lower in patient samples (Fig 2). We found no differences between sex, age, treatment (such as blood transfusion or iron chelation therapy) and associated disease.

### Protein levels of Prdx1, Trx1, Srx1, and Sod1 are higher in erythrocytes of BTI patients

The difference in the transcriptional profile revealed an important regulatory role of these enzymes in patients, which may be related to the pathophysiology of the disease. However, post-transcriptional changes may also account for different protein contents of these enzymes. Therefore, we have set out to determine the protein abundance of these enzymes in red cells of those patients.

**Fig 3:**
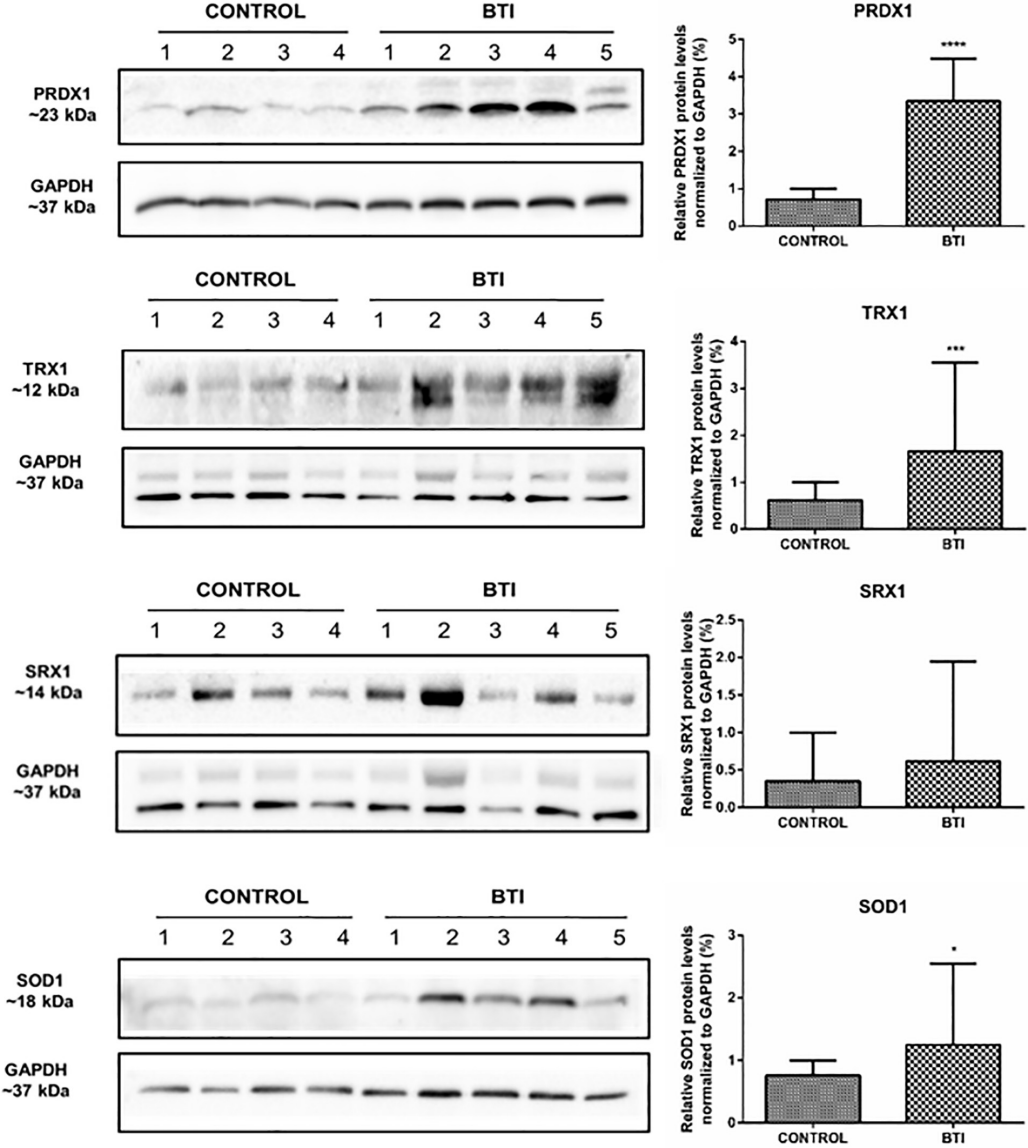
Representative western blot of Prdx1, Trx1, Srx1 and Sod1 expression in the erythrocyte cell lysate of beta-thalassemia intermedia patients compared to healthy individuals. Protein levels were measured in mature cell erythrocyte lysate from 10 patients and 8 healthy subjects. Samples were separated in a 12% (w/v) reducing SDS–PAGE using 50 μg of total protein from each sample. The intensity of the bands was measured using GAPDH as the endogenous reference. Quantitative analyzes were performed by densitometry using ImageJ Software [35]. The results are presented as mean and standard error (± SEM), and are representative of two independent experiments (*p< 0.05), (***p< 0.0001).

In agreement with mRNA levels, our data showed a statistically significant increase in protein production of Prdx1, Trx1, and Sod1 (Fig 3). Despite the fact that Srx1 showed no statistically significant difference, the protein contents in erythrocytes of BTI patients were approximately 91% higher than in healthy individuals (Fig 3). In contrast, protein levels of Prdx2, Prdx6, TrxR1, Cat1, and Gpx1 did not show significant differences between patients and controls (data not shown). As for transcription analysis, we found no differences between sex, age, treatment (such as blood transfusion or iron chelation therapy) and associated disease.

### Protein content of Prdxs 1 and 2 displays opposite patterns in BTM and intermedia BTI patients

**Fig 4:**
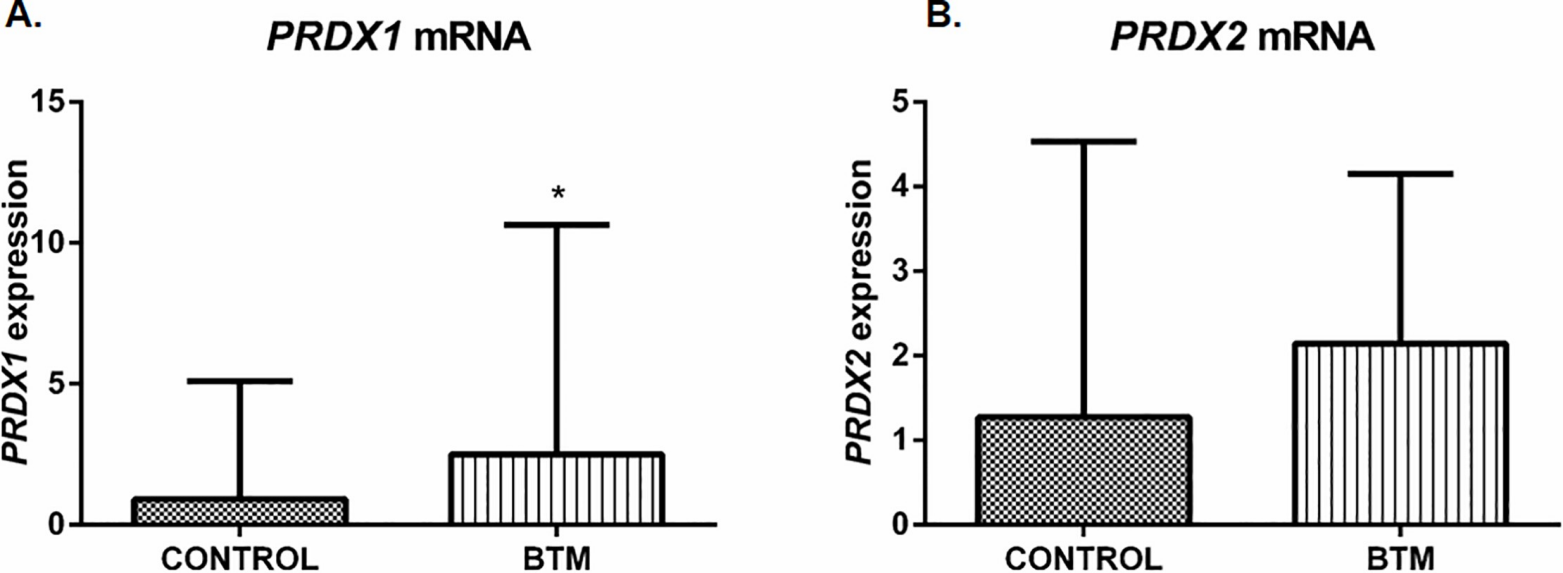
Transcription analysis of *Prdx1* and *Prdx2* gene expression in reticulocytes of BTI and BTM patients. RT-qPCR was carried out using the primers for each gene as described in S1 Table. mRNA abundance for each gene was normalized to *Bac* as endogenous reference. Results are presented as mean and standard error (± SEM). Statistical significance: *p < 0.05.

**Fig 5:**
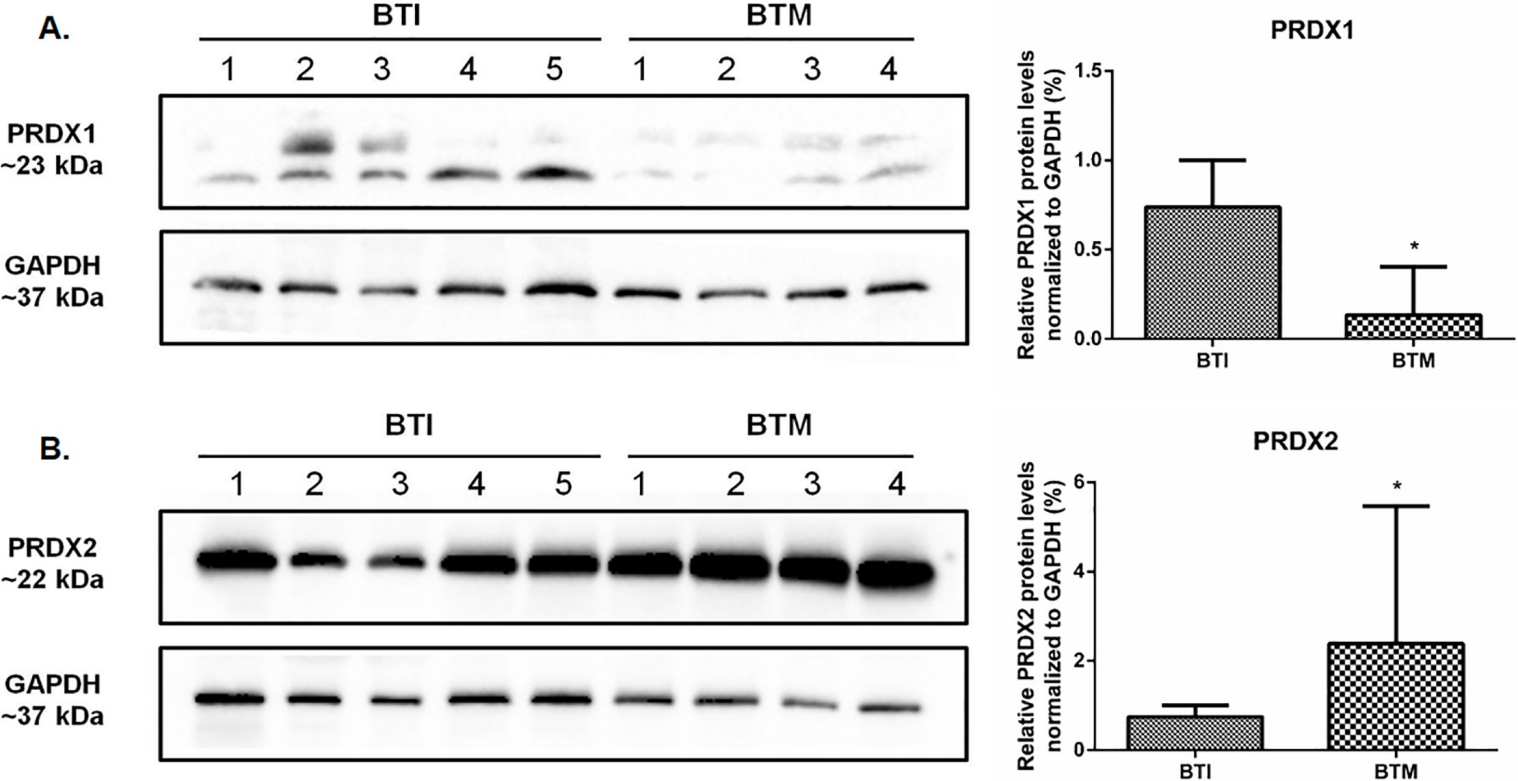
An opposite level of Prdx1 and Prdx2 was observed between beta thalassemia intermedia and major patients. Protein levels were measured using 50 μg of total protein from each sample running in a 12% (w/v) reducing SDS–PAGE. The intensity of the bands was measured using GAPDH as endogenous reference. Quantitative analyzes were performed by densitometry using ImageJ Software [35]. Results are presented as mean and standard error (± SEM), and are representative of two independent experiments. Statistical significance: *p < 0.05.

Since an increase of Prdx1 in βthal intermedia (BTI) was observed, we compared its expression with βthal major (BTM) patients, in which a high content of ROS was also observed. We showed that the transcription of this enzyme was also upregulated in this group (Fig 4A). However, we found a surprising striking reduction in the expression of this enzyme in BTM when compared to BTI patients (Fig 5). As aforementioned, Prdx2 content is the same among BTI patients and healthy individuals, although a decrease in mRNA for this enzyme was observed in BTI. An additional comparison of Prdx2 expression between BTI and BTM showed no difference at transcription levels of BTM patients (Fig 4B), but we found a high increase at protein levels (Fig 5) as previously described in the literature. Since Prdx1 and Prdx2 were very similar (about 90% in amino acid sequence) we used a recombinant human protein to test unspecific ligation among them that was still not observed (data not show). Therefore, our data provide an unreported finding of contrasting accumulation of these two Prdx enzymes in tow clinically relevant phenotypes of βthal.

### Hyperoxidation of 2-Cys Prdxs is increased in BTM patients

**Fig 6:**
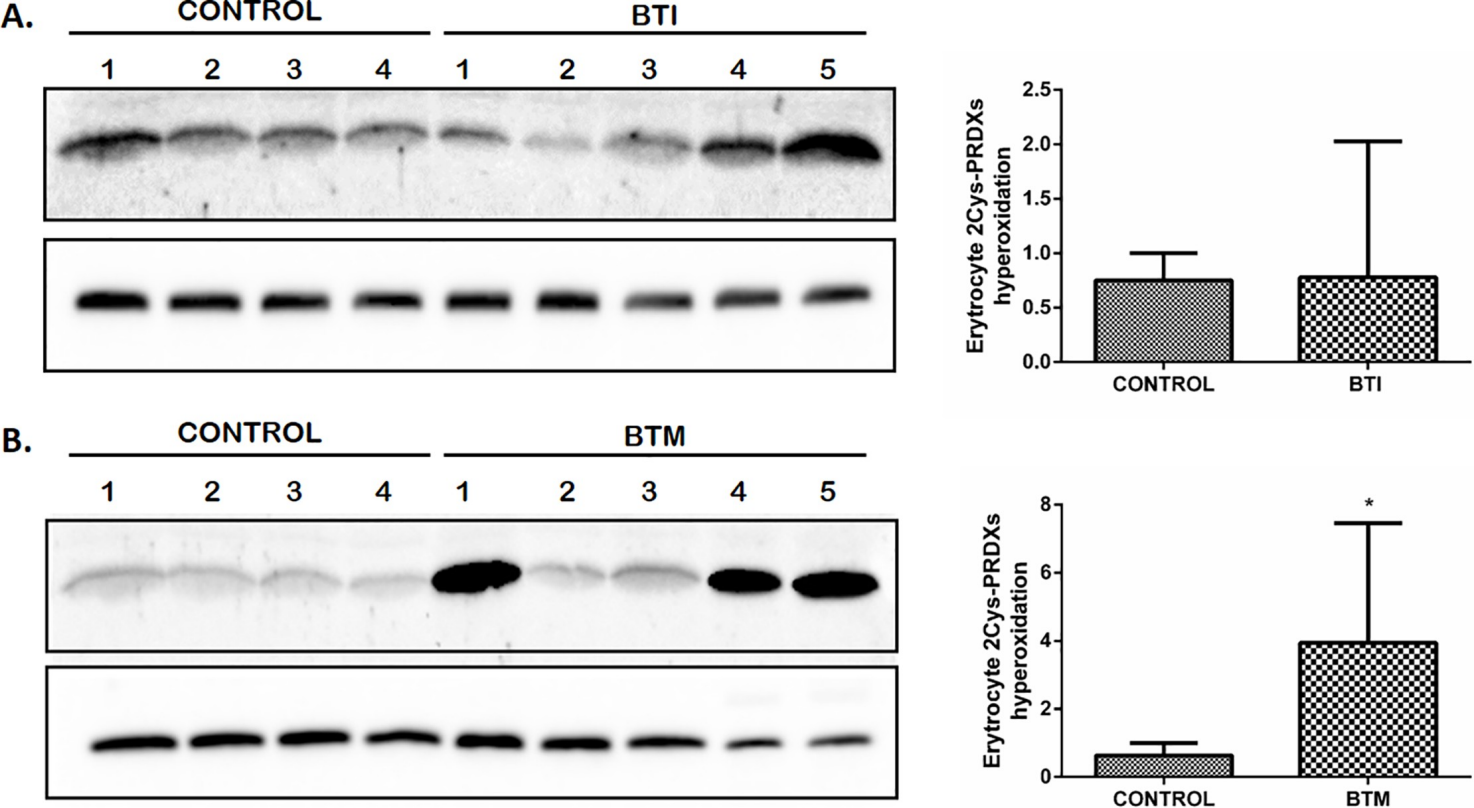
Hyperoxidation of 2Cys-Prdx is altered in patients BTM. Representative western blot for hyperoxidation state of 2CysPrdx. Erythroid cells of BTI (A) and BTM (B) patients were lysed in the presence of 200 uM of Nethylmaleimide (NEM) to prevent further sample oxidation. Hyperoxidation levels were measured using 50 μg of total protein from each sample running in a 12% (w/v) reducing SDS–PAGE. The intensity of the bands was measured using GAPDH as the endogenous control. Quantitative analyzes were performed by densitometry using ImageJ Software [35]. Results are presented as mean and standard error (± SEM). Statistical significance: *p < 0.05.

Prdx2 protein levels were not affected in BTI patients or healthy individuals. However, high levels of this protein were observed in BTM patients. On the other hand, there is an increase in Prdx1 content in BTI when compared to BTM. We have evaluated the hyperoxidation state of these enzymes since both proteins are 2-Cys Prdx and the active site of cysteine could be hyperoxidized to Cys-SO_2_H^-^ or to Cys-SO_3_H^-^, resulting in their catalytic inactivation [36, 37]. For these analyses, the erythroid cells were lysed in the presence of N-ethylmaleimide (NEM) to avoid artifactual oxidations. Our results showed that the 2-Cys Prdxs hyperoxidation was observed only for BTM patients, and it is noteworthy that this increase was higher in 3 out of 5 analyzed BTM patients (Fig 6B).

### Gene expression of Nrf2 / Keap1 complex is differentially regulated in BTI

**Fig 7:**
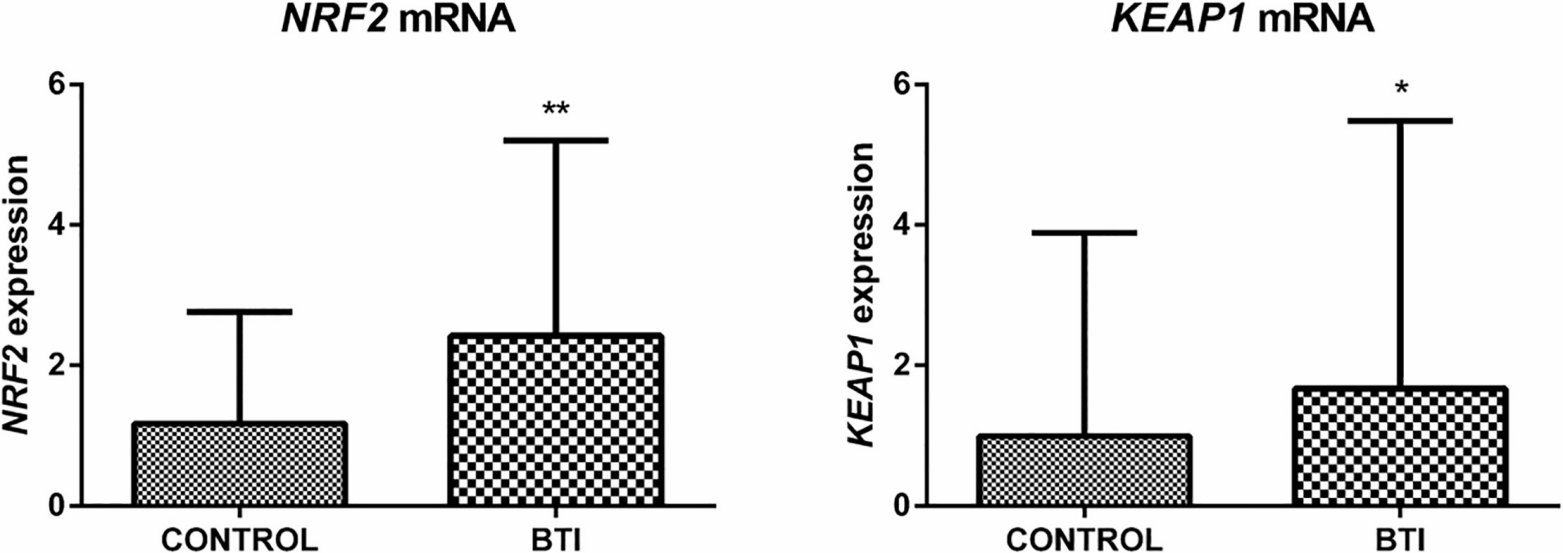
Analysis of the gene expression of *Nrf2* and *Keap1* in reticulocytes of BTI patients (n = 15) and healthy individuals (n = 16). RT-qPCR analyzes showed an increase in the gene expression of *Nrf2* (approx. 3 fold change) and *Keap1* (approx. 2 fold change) in the reticulocytes of the BTI group when compared to the control group. Primers used for these analyzes are described in S1 Table. mRNA abundance for each gene was normalized to *Bac* as the endogenous reference. Results are presented as mean and standard error (± SEM). Statistical significance: *p < 0.05, **p < 0.001.

The regulation of antioxidant systems and the cellular response to oxidative stress involves several pathways. Nuclear factor (erythroid-derived 2)-like 2 (Nrf2)/ Kelch like ECH-associated protein 1 (Keap1) system regulates the expression of several antioxidant enzymes, such as Prdx1, Prdx2, Trx1, Srx1 and Sod1 [38–44]. The *Nrf2- Keap1* system is a redox switch based in cysteines. Under oxidative stress Keap1 forms a homodimer as a consequence of the oxidation of an intermolecular disulfide (residue Cys151), releasing the Nrf2, which is able to activate the transcription of antioxidants proteins [45]. Therefore, since the levels of oxidants are very high in BTI (Fig 1) we analyzed the gene expression of the *Nrf2* and *Keap1* in reticulocytes of BTI patients and healthy individuals. Both genes were upregulated in reticulocytes of BTI patients (Fig 7).

## Discussion

Several studies have already described the generation of ROS in erythrocytes and their significance in the physiopathology of beta-thalassemia. Some of them have shown that this increase is higher in BTM than BTI, both in plasma and erythrocytes [46, 47]. Besides corroborating these findings, our results also described increased levels of ROS in mononuclear cells and neutrophils of these patients, indicating that oxidative damages are not restricted to erythroid cells (Fig 1). The oxidative stress impairs the phagocytic function and triggers premature senescence of T lymphocytes, which in turn, impairs the immune system function [12, 48]. A study by Amer and Fibach showed that beta-thalassemic neutrophils undergoing chronic oxidative stress exposure present a reduction in their ability to induce the respiratory burst, therefore compromising their antibacterial function through innate immune response. In this sense, an increase of ROS in these cells could aggravate the susceptibility of these patients to recurrent infections [49].

The performance of the enzymatic antioxidant defense system is of paramount importance for the maintenance of the erythrocytes, placing Prdxs in prominence due to their abundance and reactivity towards hydroperoxides [26, 50–53]. Here, we showed that the expression of *Prdx1* gene and protein content was increased in BTI reticulocytes and erythrocytes, when compared to healthy controls (Figs 2 and 3) and, despite an upregulation for this enzyme found at transcription level in BTM patients (Fig 4), the protein content was only higher in BTI patients (Fig 5). The importance of Prdx1 for the survival of RBC was previously demonstrated in mice [22]. We speculate that, in BTM patients, the mRNA for this enzyme, although up-regulated, was not able to produce sufficient amounts of protein, because the high amounts of ROS found in these patients could increase the oxidative damage leading to degradation [54]. On the other hand, the increase in the production of Prdx1 can cooperate with Prdx2 in the reduction of H_2_O_2_ in erythrocytes of BTI patients.

Also regarding Prdx1, previous studies in different cell types have demonstrated that, concomitantly with peroxidase activity, this enzyme and its physiological reductant Trx1 have anti-apoptotic functions in the cytoplasm through direct or indirect interaction with important apoptotic regulators induced by oxidative stress, such as ASK1, p66^shc^ and JNK [55–58]. Our results also evidenced significantly elevated levels of *Trx1* in BTI (Figs 2 and 3), indicating that the increase of these two proteins may represent an additional mechanism of cellular protection in the defense against induced cell death by oxidative stress. Additionally, Prdx1 was also involved in host defense against infection from microorganisms inducing Interleukin 12 (IL-12) and Nitric Oxide (NO) production. This may be also the case for BTI patients, whose inflammatory processes are evident and need to be considered in the management of beta thalassemic patients. However, quantification of Interleukin 12 (IL-12) and Nitric Oxide (NO) and the relationship with Prdx1 in BTI awaits further experimentation. The differential expression observed for Prdx1 between BTI and BTM, possibly act as a phenotypic modulator of the severity of the disease. Nonetheless, further studies are needed to better establish this relationship.

The importance of Prdx2 in the maintenance of red blood cells was demonstrated in a study that showed high levels of ROS, increased Heinz bodies formation and severe hemolytic anemia in a Prdx2^-/-^ mice. Thus, it is likely that Prdx2-regulated redox balance in erythrocytes is closely associated with hematological pathologies, such as reduced erythrocyte lifespan and hemoglobin instability [25]. Additionally, a study by De Franceschi et al. demonstrated an increase in the levels of this enzyme when analyzing erythroid cultures of beta thalassemia with the CD39 mutation (β0) [59]. Another study using two models of beta-thalassemic mice with different severities carried out by this same group observed that the increase in PRDX2 levels is related to the severity of the disease [28]. Our results indicated reduced mRNA levels of this enzyme in reticulocytes of BTI patients (Fig 2), but with no differences at the protein level (data not shown). The data suggest that in these patients the peak of production of Prdx2 occurred in previous stages of erythroid development or the existence of post-transcriptional processes that have not yet been elucidated. In BTM, the levels of Prdx2 are highly increased when compared to BTI (Fig 5).

Although Prdx2 levels are higher in BTM, the cysteine hyperoxidation of these enzymes is also higher in these patients (Fig 6). Some studies revealed that hyperoxidation of Prdxs may be important to increase the levels of reduced Trx1, relevant to maintain processes such as repair pathways that are crucial to cell survival [60–62]. Additionally, since that Prdx2 was found associated with the membrane erythrocyte and can act as a molecular chaperone when in hyperoxidized state [19], the hyperoxidation may play a role as an alternative protection of the membrane proteins to enhance the life span of cell [23, 63]. In addition, Prdx2 is approximately 80-fold more expressed in erythrocytes than Prdx1 and is the first peroxiredoxin to be active after an increase in ROS inside the cells to protect from oxidative damage. Moreover, Prdx2 are also easily retroreduced than Prdx1 [64]. Therefore, the overoxidized forms detected by western blot may largely correspond to Prdx2. However, since the generation of ROS is continuously increasing in these cells, this mechanism could not work adequately in BTM. Although the ROS generation is also high in BTI cells, the augment of Prdx1 expression together with the existing Prdx2 could contribute to the detoxification of hydroperoxides, thereby minimizing hyperoxidation and prolonging the lifespan of RBC in these patients.

In relation to the other antioxidant enzymes, we observed an increase in transcription and protein expression of Sod1 in BTI when compared to healthy individuals. Previous studies have reported that Sod1-deficient mice had severe hemolytic anemia among other alterations, highlighting the importance of this enzyme for erythrocytes, which are constantly exposed to high concentrations of superoxide anion generated by autoxidation of hemoglobin [10, 65]. The increase of *Sod1* mRNA levels and protein expression reinforce the role os Sod1 in βthal disease. Sod1 increase may be the result of compensatory mechanisms in response to high levels of ROS observed in BTI (Fig 1).

Previous studies have shown that Prdxs and other antioxidants enzymes can also be regulated by the Nrf2/Keap1 system in different cell types [38, 41, 66–69]. Our results revealed an increase in mRNA levels of the Nrf2/Keap1 complex in reticulocytes of BTI (Fig 7). Under redox homeostasis, Nrf2 is located in the cytoplasm forming an inactive complex with Keap1 [70]. However, during oxidative stress, Nrf2 is phosphorylated by protein kinases such as PKCδ that dissociates from Keap1 and migrates to the nucleus, activating the transcription of antioxidants enzymes [71, 72]. Additionally, oxidative stress caused by H_2_O_2_ or reactive nitrogen species, is able to promote the formation of a Keap1 intermolecular disulfide, also allowing Nrf2 releasing [73]. Although both genes have been upregulated in reticulocytes, the increase in expression was more pronounced in Nrf2 (approx. 3 fold change) than in Keap1 (approx. 2 fold change). Additionally, the expression of PKCδ was also increased in the early stages of thalassemic culture cells (data not shown), suggesting an augment in the phosphorylation of Nrf2/Keap1 complex. Together with the increase of ROS in these cells (Fig 1) which may results in cysteine Keap1 oxidation, both mechanisms could contribute to the formation of free Nrf2 and to the upregulation of antioxidants enzymes such as Prdx1 and Sod1 [74]. In addition to its activity as a regulator of several antioxidants, Nrf2 also regulates the transcription of the beta and gamma-globin gene and is an important regulatory element in the control of heme and globin synthesis, promoting balance in the production of these two components [75].

**Fig 8:**
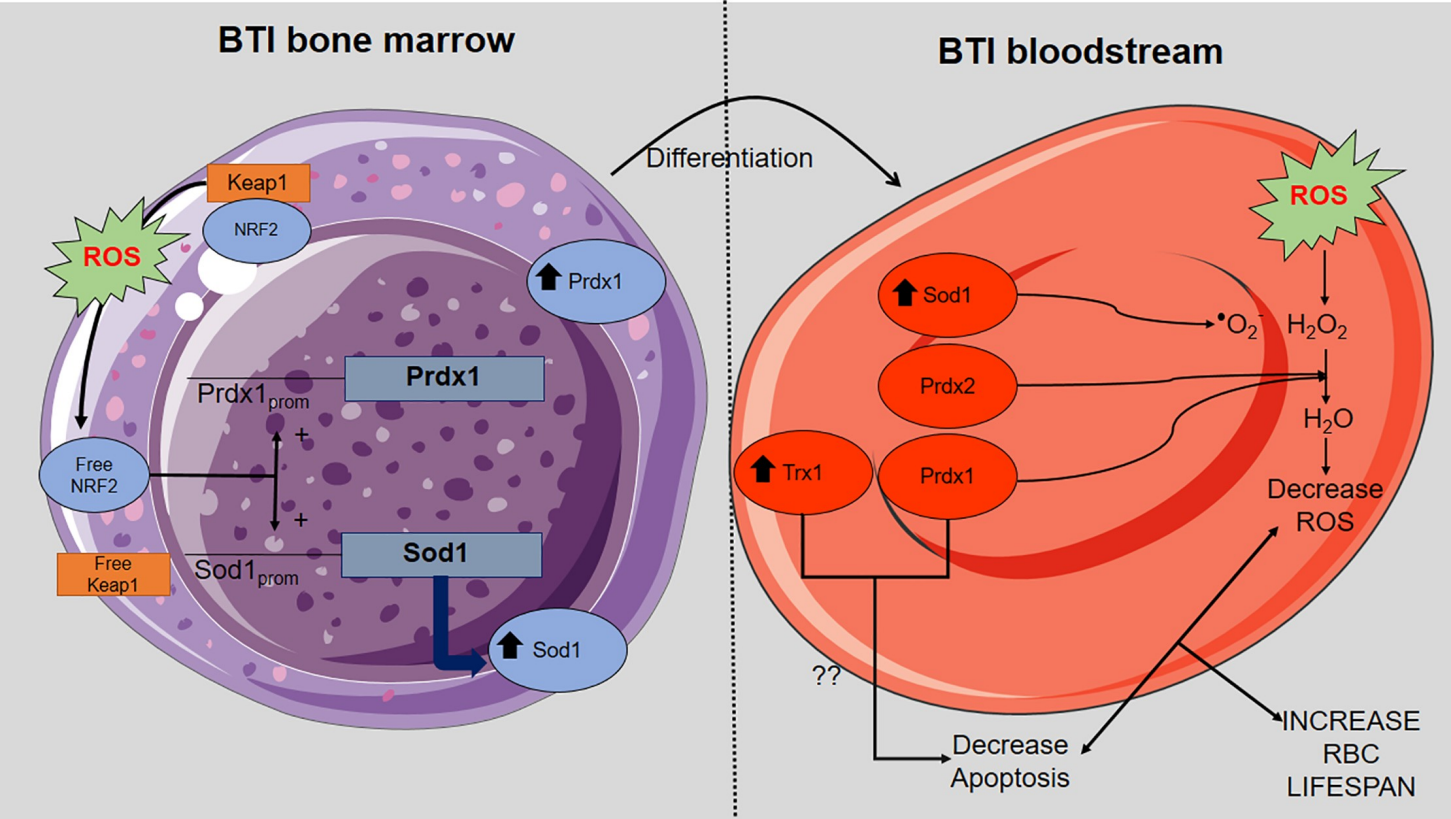
Model of interaction between Prdx1 and Prdx2 in the detoxification of hydroperoxides of BTI RBCs. An increase in ROS production during the development of RBCs results in Keap1 oxidation and consequent liberation of free Nrf2, contributing to the upregulation of Prdx1 and Sod1. In the bloodstream the upregulation of Prdx1 can act together with Sod1 and Prdx2 in the detoxification of ROS. In addition, Prdx1 associated with Trx1 leads to decrease in apoptosis. Altogether, these processes contribute to increase BTI RBCs lifespan.

Overall, our results describe for the first time a wide panorama of the regulation of pathways related to the control of oxidative stress in BTI erythroid cells. A model summarizing our results based on Prdx1 and Prdx2 functions in BTI patients is depicted in Fig 8.

We have also added other players in this process to better comprehend this important disease.

## Supporting information

S1 Fig Leukocyte lamina and reticulocytes smear. A) During the process of reticulocytes separation there is a phase in which leukocytes are precipitated, for comparative purposes, a lamina was prepared using precipitate. B) To check for possible contamination by leukocytes during reticulocyte extraction, a lamina was prepared with the smear of the resulting pellet after extraction. Laminas A and B were stained with Panotic, a dye used for staining leukocytes. The results obtained showed the presence of a negligible amount of leukocytes in lamina B, discarding the pellet contamination. The laminas shown in the images C and D were also prepared with smear from the pellet obtained with reticulocyte extraction performed according to the protocol described above and stained with brilliant cresyl blue, used to stain reticulocytes. Figure C enables the visualization of RNA remnants that precipitates forming beads. These granules disappear when the reticulocyte completes differentiation into mature erythrocytes.
(TIF)

S1 Table Primer sequences. (PDF)
