# Supplementary figures and images for: Global analysis of erythroid cells redox status reveals the involvement of Prdx1 and Prdx2 in the severity of beta thalassemia
Source: PLoS One. 2018 Dec 6;13(12):e0208316. doi: 10.1371/journal.pone.0208316 (PMC6283586; doi:10.1371/journal.pone.0208316)

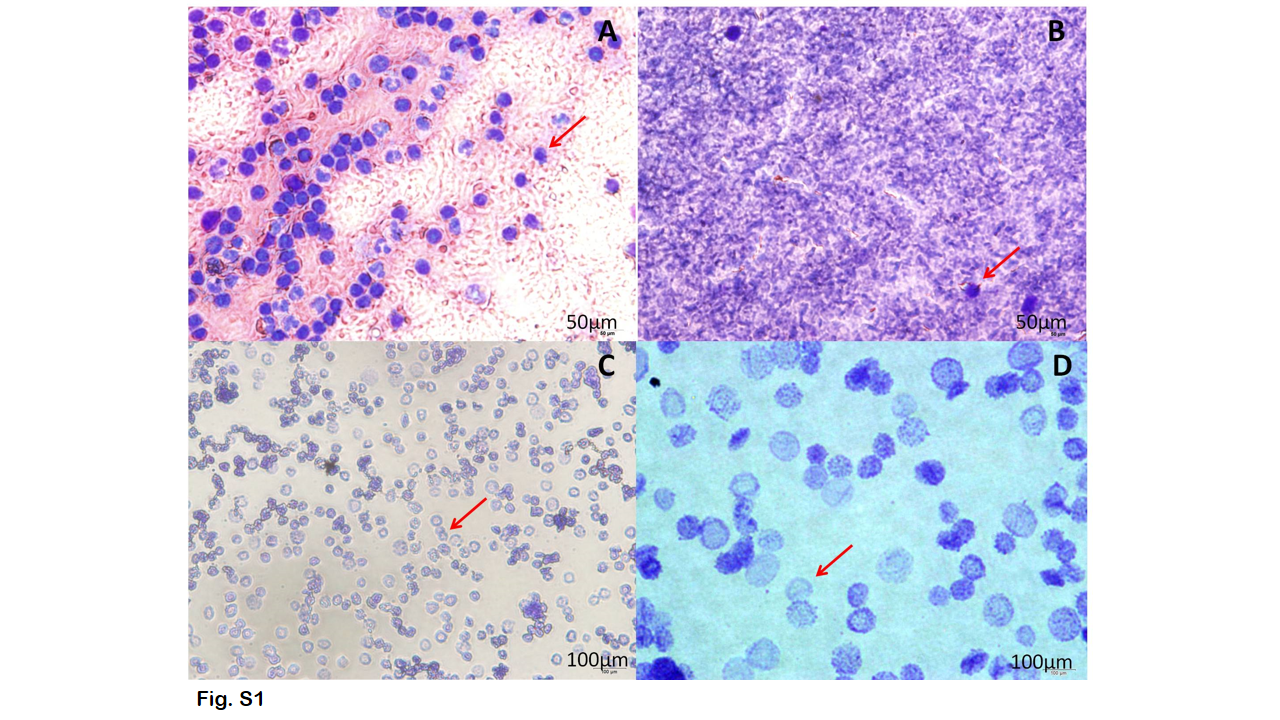

Supplement: S1 Fig — A) During the process of reticulocytes separation there is a phase in which leukocytes are precipitated, for comparative purposes, a lamina was prepared using precipitate. B) To check for possible contamination by leukocytes during reticulocyte extraction, a lamina was prepared with the smear of the resulting pellet after extraction. Laminas A and B were stained with Panotic, a dye used for staining leukocytes. The results obtained showed the presence of a negligible amount of leukocytes in lamina B, discarding the pellet contamination. The laminas shown in the images C and D were also prepared with smear from the pellet obtained with reticulocyte extraction performed according to the protocol described above and stained with brilliant cresyl blue, used to stain reticulocytes. Figure C enables the visualization of RNA remnants that precipitates forming beads. These granules disappear when the reticulocyte completes differentiation into mature erythrocytes. (TIF) [file pone.0208316.s001.TIF]
