# Supplementary material for: Global analysis of erythroid cells redox status reveals the involvement of Prdx1 and Prdx2 in the severity of beta thalassemia
Source: PLoS One. 2018 Dec 6;13(12):e0208316. doi: 10.1371/journal.pone.0208316 (PMC6283586; doi:10.1371/journal.pone.0208316)
Supplement: S1 Table — (PDF) [file pone.0208316.s002.pdf]

Table S1. Primer sequences

Gene	Accession number	Forward primer	Reverse primer
Beta Actin (BAC)	NM_001101.3	5’-CAAGCAGGAGTATGACGAGTC-3’	5’- GCCATGCCAATCTCATCTTG-3’
Hypoxanthine Phosphoribosyltransferase 1 (HPRT1)	NM_000194.2	5´- AGATGGTCAAGGTCGCAAG -3´	5´- GTATTCATTATAGTCAAGGGCATATCC-3´
Peroxiredoxin 1 (PRDX1)	NM_002574.3	5'-TGTAAATGACCTCCCTGTTGG-3'	5'-TATCACTGCCAGGTTTCCAG-3'
Peroxiredoxin 2 (PRDX2)	NM_005809.5	5'-CTGTTAATGATTTGCCTGTGGG -3'	5'-TGGGCTTAATCGTGTCACTG-3'
Peroxiredoxin 3 (PRDX3)	NM_006793.4	5’- TCCCACTTTAGCCATCTTGC-3’	5’- GTAGTCTCGGGAAATCTGCTTAG-3’
Peroxiredoxin 4 (PRDX4)	NM_006406.1	5’- CGTTTGGTTCAAGCATTCCAG-3’	5’- CTTTTGGCGACAGACTTGAAG-3’
Peroxiredoxin 5 (PRDX5)	NM_012094.4	5’- AGAAGGGTGTGCTGTTTGG-3’	5’- TCATTAACACTCAGACAGGCC-3’
Peroxiredoxin 6 (PRDX6)	NM_004905.2	5'-CACGACTTTCTGGGAGACT-3'	5'-GGGCAATCAACTTAACATTCCTC-3'
Catalase (CAT)	NM_001752.3	5'-TGAATGAGGAACAGAGGAAACG-3'	5'-GTACTTGTCCAGAAGAGCCTG-3'
Superoxide Dismutase 1 (SOD1)	NM_000454.4	5'-GGGCAAAGGTGGAAATGAAG-3'	5'-CAGCTAGCAGGATAACAGATGAG-3'
Glutathione Peroxidase (GPX1)	NM_000581.2	5'-TTCCAGACCATTGACATCGAG-3'	5'-CACCCTCATAGATGAAAACCCC-3'
Sulfiredoxin (SRX)	NM_080725.2	5'-AGCATCCACACCAGACTTG-3'	5'-ACCCCTGCTATCCCTTCTG-3'
Thioredoxin 1 (TRX1)	NM_003329.3	5’-TGGTGAAGCAGATCGAGAGCAAAGA-3'	5’-ACCACGTGGCTGAGAAGTCAACTA-3’
Thioredoxin Reductase 1 (TRXR1)	NM_003330.3	5’-CAACATTGTCTGTGACCAAGC-3’	5’-AACCCTCTAGAAAACTTCGCC-3’
Nuclear Factor Erythroid 2-Related Factor 2 (NRF2)	NM_006164.4	5’- GCTACGTGATGAAGATGGAAAAC-3’	5’- AGCTCAGAAAAGGTCAAATCCTC-3’
Kelch-like ECH-associated protein 1 (KEAP1)	NM_012289.3	5’- AACAGAGACGTGGACTTTCG-3’	5’- GTGTCTGTATCTGGGTCGTAAC-3’
Protein kinase C delta (PKCδ)	NM_006254.3	5’- TGGAAAAGCGGAGGTTGG-3’	5’- ATGGAGTCGATGAGGTTCTTG-3’
ETS Proto-Oncogene 1, Transcription Factor (ETS1)	NM_001143820.1	5’- ATCATCCACAAGACAGCGG-3’	5’- GTTTCCCCAGCCCCTTC-3’
ETS Proto-Oncogene 2, Transcription Factor (ETS2)	NM_005239.5	5’- TCAGACAAATCCTGCCAGTC-3’	5’- GCTTCTCGTAGTTCATCTTGGG-3’
